# A timely method for post-disaster assessment and coastal landscape survey using drone and satellite imagery

**DOI:** 10.1016/j.mex.2023.102065

**Published:** 2023-02-06

**Authors:** Marcelo Cancela Lisboa Cohen, Adriana Vivan de Souza, Kam-biu Liu, Qiang Yao

**Affiliations:** a Graduate Program of Geology and Geochemistry, Federal University of Pará, Av. Perimentral 2651, Terra Firme, Belém, PA 66077-530, Brazil; b Department of Oceanography and Coastal Sciences, College of the Coast and Environment, Louisiana State University, Baton Rouge, LA 70803, United States of America

**Keywords:** Drone, Satellite imagery, Landscape dynamics, Natural disaster, Spatial-temporal analysis, Spatial-temporal analysis using drone and satellite imagery

## Abstract

To mitigate floods and storm surges, coastal communities across the globe are under the pressure of high-cost interventions, such as coastal barriers, jetties, and renourishment projects, especially in areas prone to hurricanes and other natural disturbances. To evaluate the effectiveness of these coastal projects in a timely fashion, this methodology is supported by a Geographic Information System that is instaneously fed by regional and local data obtained shortly (24 h) after the disturbance event. Our study assesses the application of 3D models based on aerophotogrammetry from a Phantom 4 RTK drone, following a methodological flowchart with three phases. The Digital Elevation Models (DEMs) based on aerophotogrammetry obtained from a Phantom 4 RTK drone presented a low margin of error (± 5 cm) to dispense Ground Control Points. This technique enables a rapid assessment of inaccessible coastal areas due, for instance, to hurricane impacts. Evaluation of DEMs before and after the disturbance event allows quantifying the magnitudes of shoreline retreat, storm surges, difference in coastal sedimentary volumes, and identifying areas where erosion and sediment accretion occur. Orthomosaics permit the individualization and quantification of changes in vegetation units/geomorphological areas and damages to urban and coastal infrastructure. Our experience monitoring coastal dynamics in North and South America during the last decade indicates that this methodology provides an essential data flow for short and long-term decision-making regarding strategies to mitigate disaster impacts.

• Permanent and regional monitoring with spatial-temporal analysis based on satellite/aerial images and lidar data prior to the event.
• Local DEMs based on drone aerophotogrammetry after the event.
• Integration of regional and local planialtimetric/environmental data.

## Graphical abstract

**Figure:**
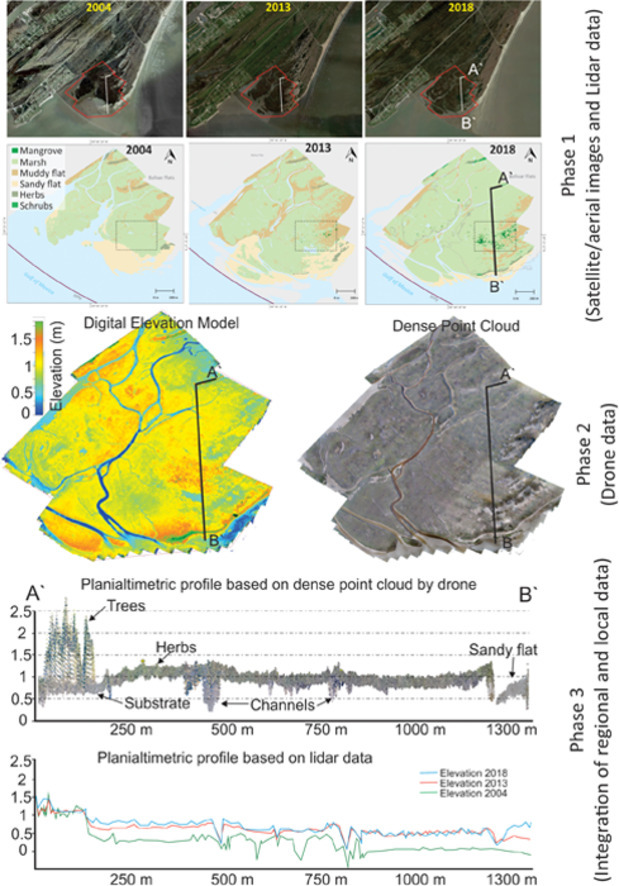
The DEMs and planialtimetric and vegetation profiles of the Bolivar Flats Shorebird Sanctuary, Texas, USA

Specifications table

**Table:** 

Subject area:	Earth and Planetary Sciences
More specific subject area:	*Remote Sensing*
Name of your method:	*Spatial-temporal analysis using drone and satellite imagery*
Name and reference of original method:	*N/A*
Resource availability:	*N/A*

## Method details

### Rationale

Currently, approximately 40% of the world's population lives within 100 km of the coast. However, many coastal zones around the globe are suffering from the combined effects of flooding, subsidence, rising sea levels, and particularly storms. Intense storms are one of the most devastating earth surface processes that cause trillion dollars of economic loss and over 10,000 fatalities across the globe each year (according to the United Nations Office for Disaster Risk Reduction). Moreover, anthropogenic activities have drastically changed the coastal landform since the 19th century. Using the Galveston Bay as an example, jetties and levees were constructed to stop the natural overbank flooding, and numerous waterways and canals were dredged to support the shipping and petroleum industries [1]. These activities have caused significant saltwater intrusion, wetland fragmentation, and erosion in the Galveston area. In recent decades, many coastal restoration and engineering projects were undertaken to combat these natural and anthropogenic disturbances worldwide [2,3]. Can they successfully restore the coastal stability? This question has not been adequately addressed, yet it has significant scientific implications. Thus, establishing a timely method for post-disaster assessment and coastal landscape survey have become a focus of research and concern for coastal scientists and stakeholders around the world.

To date, remote sensing is still the most common method of studying coastal landform dynamics [4,5]. This method is time-tested and relies on high resolution satellite imagery such as lidar and QuickBird. However, satellite imagery is not always available because of blockage by clouds and trees. Moreover, ground truthing is another obstacle faced by many remote sensors. In addition, due to the refreshing rate of satellite imagery, an immediate survey of the area of interest is not always feasible, especially for a time-sensitive situation such as a post-disaster assessment. In this study, we present a step-by-step procedure of an optimized spatial-temporal analysis using a combination of satellite and drone imagery to conduct post-disaster assessment and coastal landscape survey in a timely fashion. This methodology and Ground Contol Points are assocaited with a study conducted in Bolivar Flats, Texas [6].

## Required materials and instruments

### General materials

1. Ruler (1–5 m) for ground elevation verification
2. Black rubber mats (1 m^2^) with a yellow cross to mark the ground control points (GCPs) for drone
3. Clipboard
4. Camera with georeferencing
5. Two-way radio communication device
6. Wind speed meter

### Key instruments (Fig. 1)

1. DJI Phantom 4 drone Real-Time Kinematic with a digital 4K/20MP (RGB).
2. D–RTK 2 Mobile Station with differential corrections transmitted in real-time from a network
3. Antenna Trimble DA2 Catalyst (GNSS) (accuracy ± 1 cm - Real-Time Kinematic)
4. Electronic Self Leveling Horizontal Rotary Laser
5. Computer (>16 GB Ram, >1 TB SSD).
6. External hard disk (> 5 TB)

### Software

1. Google Earth Pro
2. Agisoft Metashape Professional 1.8.4
3. Global Mapper V22.1.2

## Procedures

The techniques described in this method strictly followed a methodological flowchart with three phases: (1) Obtain permanent and regional spatial-temporal analysis based on satellite/aerial images and lidar data prior to the disturbance event; (2) Construct local DEMs based on drone aerophotogrammetry and ground control points after the disturbance event; (3) Intergrade regional and local planialtimetric/environmental data in a Geographic Information System (GIS) (Fig. 2). This flow-chart enabled the sequential integration of planimetric and altimetric records based on lidar, satellite and drone data that feed a GIS in the Global Mapper 22.1.2 Software. This GIS facilities the assessment of the impact, pin-points the landing and take-off points for the drone, maps the study area, critically analyzes the results, and evaluates the economic loss. This procedure provides a robust spatial-temporal analysis within a reasonable timeframe after the disturbance event. A step-by-step description of this methodological sequence is listed in the following paragraphs (Fig. 2).

### Satellite and aerial images

Aerial images, recorded in 1969 by the Texas General Land Office, and Landsat 5 images, obtained in 1990 and 1995 with a ground pixel resolution of 30 m, were used for constant and regional spatial-temporal analysis. The high-resolution spatial-temporal analysis was based on the examination of QuickBird satellite images obtained in 2004, 2007, and 2013 with a ground pixel resolution of 2.4 m (multispectral) and three bands (blue, green, and red). Such bands were chosen to match with the drone camera that only records images within the visible spectral range (blue, green, red). These images can be downloaded for free from Google Earth Engine. Satellite images were processed in the Global Mapper version 22.1.2. and orthorectified according to lidar data. And then, ground control points (GCPs) were identified in the satellite images after they were obtained. The vegetation and geomorphological features were manually classified and quantified by photo interpretation using various tools in the Global Mapper. Image processing and classification in the Global Mapper can be obtained from Ying et al. [7].

### Lidar data

The lidar point clouds were recorded in 2006 and 2016, with a vertical and horizontal accuracy of 10–15 cm and 73–100 cm, respectively [8]. These data were downloaded from the National Oceanic and Atmospheric Administration website with cloud points (previously classified) representing ground, vegetation, and water (https://coast.noaa.gov/dataviewer/#/). The lidar data were imported in LAZ format and referenced to a horizontal datum (NAD83), vertical datum (NAVD88), geoid model (EGM2008–5) and geographic projection (Lat/Lon) with horizontal units in decimal degrees. Point clouds that were not contiguous with the main landform were removed. These planialtimetric data were used to evaluate the beach barrier and wetlands dynamics. The dates of the lidar data were chosen according to their quality and availability. Details regarding the processing and classification of lidar point clouds can be obtained from Johnson et al. [9].

### Drone imagery

A DJI Phantom 4 drone Real-Time Kinematic (RTK) with a FC 6310 digital 4K/20MP (RGB) camera can record images (width) with 5472 pixels (focal length: 8.8 mm and sensor width: 12.8 mm). This camera recorded high spatial resolution images of the study area, calculated using the following Eq. (1):

(1) GSD=(Sw*H*100)/(Fr*imH),

where, GSD = Ground Sampling Distance (centimeters/pixel), Sw = sensor width of the camera (millimeters), *H* = the flight height (meters), Fr = the focal length of the camera (millimeters) and imH = the image width (pixels). Considering that the images were acquired at 100 m altitude, the GSD for the orthoimages was 2.6 cm/pixel (PIX4D, 2013).

A RTK module incorporated into the Phantom 4 RTK provides real-time, centimeter-level positioning data, improving the absolute accuracy of the images. Besides optimized flight safety and precise data collection, this drone stores satellite observation data for Post Processed Kinematics. The Phantom 4 has a TimeSync system to align the flight controller, camera and RTK module continuously. In addition, TimeSync ensures each photo uses the most accurate metadata and fixes the positioning data to the optical center of the lens, optimizing the results from aerophotogrammetric methods. We used a D-RTK 2 Mobile Station to strengthen the RTK signal and ensure maximum accuracy of the planialtimetric data obtained from aerophotogrammetry. This mobile station uses differential corrections to transmit signal in real-time via 4G, OcuSync, WiFi, and LAN from a network hub, allowing georeferencing of each photo obtained by the Phantom 4 RTK at a centimeter-level. This mobile station allows better signal reception from more satellites even when obstructions are present (see details in the DJI User Manual). The flights were carried out autonomously (90° camera angle, 90% frontal, and 75% lateral overlay, at 100 m altitude) following a single mission, and interrupted only during the battery replacement. Drone survey based on a single mission ensures an accurate photo overlay and the quality of planialtimetric data. A total of ∼2453 images were recorded to cover ∼142 ha in Nov/2018 and Nov/2022. Images were imported and processed in GeoTIFF format by the Agisoft Metashape Professional 1.8.4. A dense point cloud was generated to obtain digital models of the terrain and vegetation based on photogrammetric analysis of the drone images. The contrast of colors and elevation of point clouds enabled us to identify features indicating the ground, vegetation, water, and anthropogenic structures. Moreover, a field survey was conducted during the drone survey to confirm the topographic data, vegetation types/heights, and the limits of intertidal and supratidal zones.

### Ground control points

**Fig. 1:**
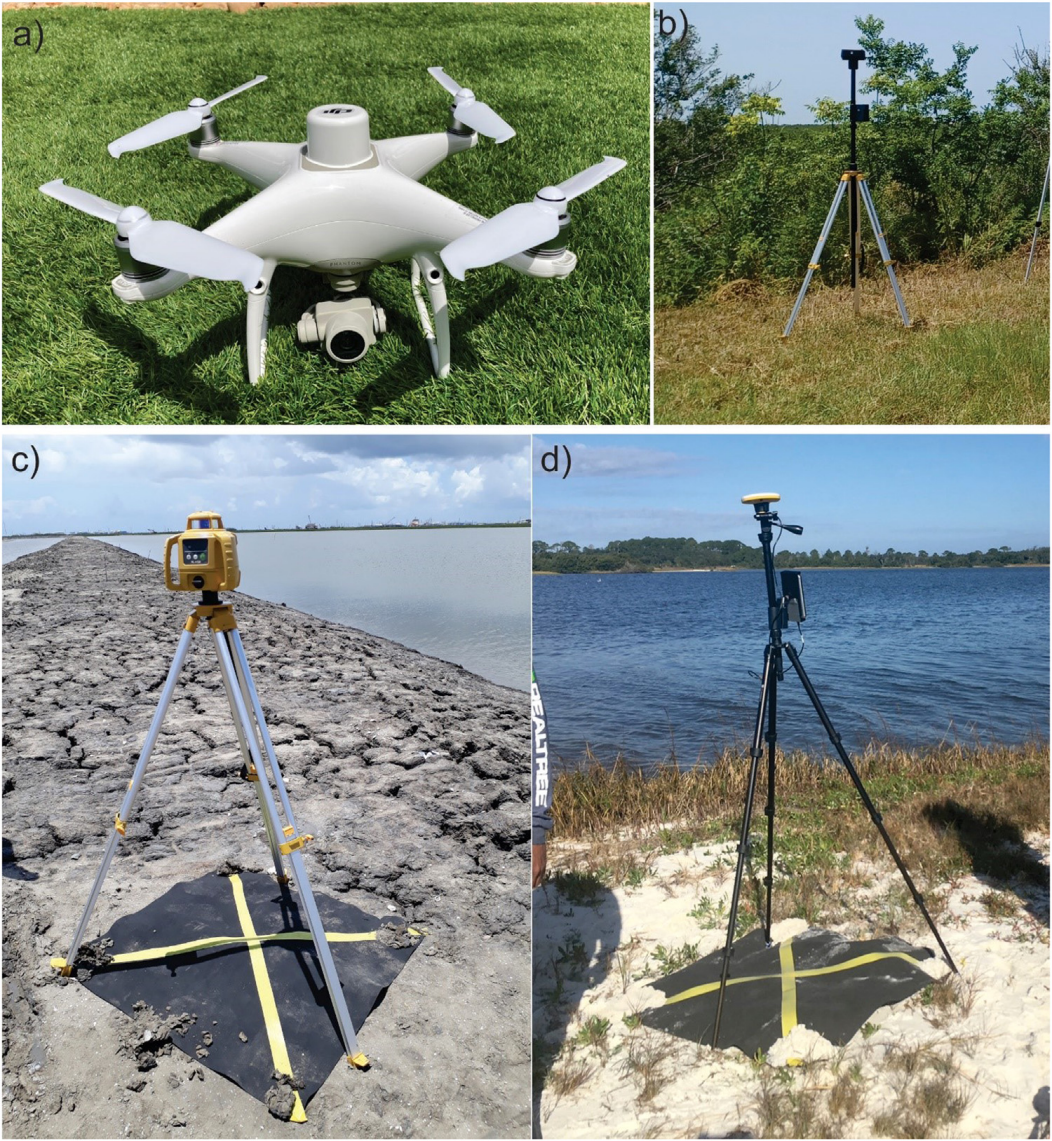
Essential instruments: (a) Drone Phantom 4 RTK, (b) D–RTK 2 Mobile Station, (c) Electronic Self Leveling Horizontal Rotary Laser, (d) Antenna Trimble Catalyst.

Planimetric and altimetric data were acquired during field trips in Nov/2018 and Nov/2022. A smartphone and a Trimble DA2 Catalyst with a differential Global Navigation Satellite System (GNSS) (accuracy ± 1 cm with the Real-Time Kinematic correction) were used to obtain absolute planialtimetric data (Fig. 1d). Such data were used as reference points for the topographic survey carried out with an electronic Self Leveling Horizontal Rotary Laser (400 m range, model Topcon RL-H5B). Once an absolute planialtimetric point based on the GNSS data was established, the Self Leveling Horizontal Rotary Laser (SLHRL) (Fig. 1c), fixed on a tripod, provides a horizontally levelled bean that defines the relative topography for other Ground Control Points (GCPs) using a small laser detector (model LS-80 L) mounted on a 5-meter aluminum scale. This device detects the laser signal and determines elevation differences between the base station and a range of points with a 2 cm vertical margin of error. The combination of the Trimble DA2 Catalyst with SLHRL allowed us to record planialtimetric data on ten ground control points (GCP) in the study area marked in the ground by the black rubber mats (1 m^2^) with yellow crosses (Fig. 1c and 1d). These GCPs were used to determine the margin of error of the digital elevation model (DEM) obtained by photogrammetry.

**Fig. 2:**
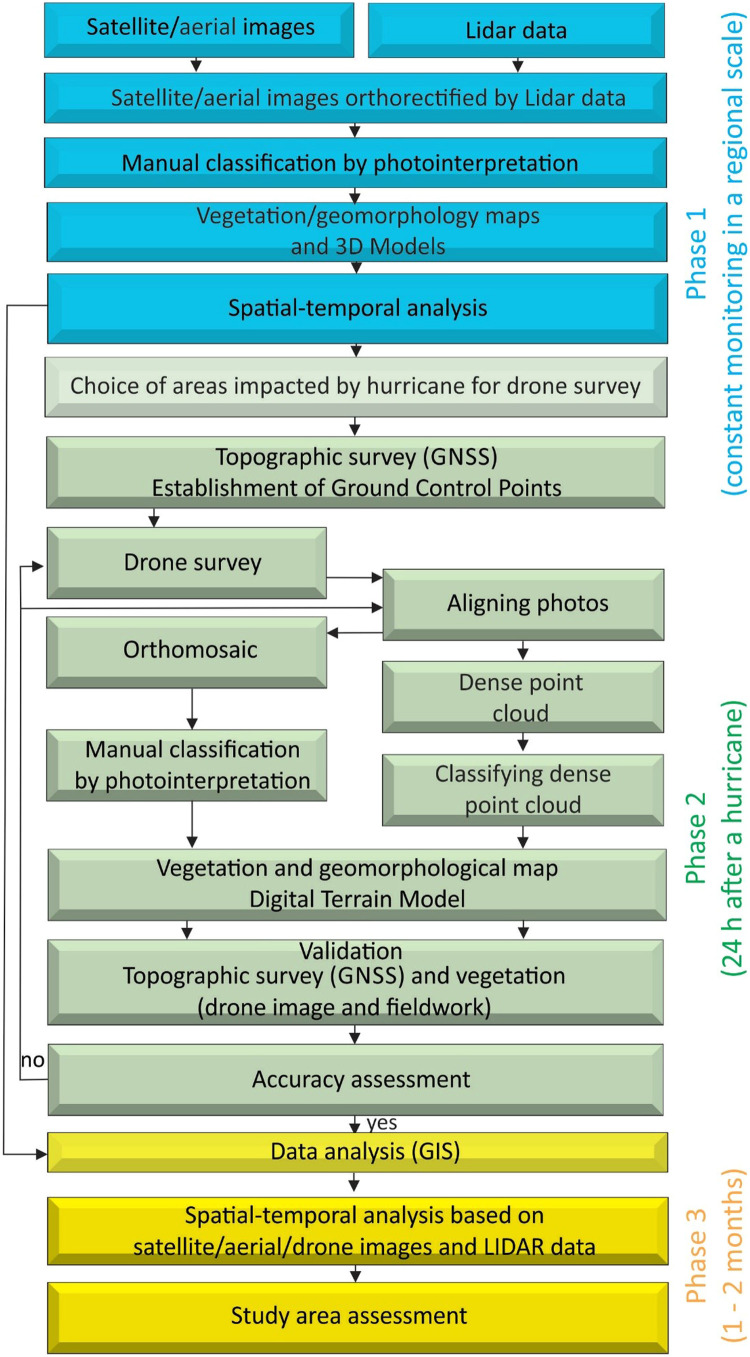
Methodology flow-chart.

### Image classification

Drone images permitted the identification of mangrove, saltmarsh, shrub, sandy flats, mud flats, water and anthropogenic interventions in the study area. These features were manually individualized and classified by photo interpretation using the Global Mapper 22.1.2 software. Multispectral digital numbers and physical and geometric characteristics of identified vegetation allowed us to individualize classes and compare them with a visual interpretation based on drone orthophotos and field observations. Panoramic aerial photos and field survey with 16 GCPs were also employed to subsidize this classification. This cross-validation data allows us to identify and establish limits for those classes with high precision. Erosion/accretion zones were automatically recorded by the comparative analysis between the digitalized limits of the mangrove, saltmarsh, and beach barrier classes identified in the following time intervals: 1969 – 2013 (aerial and satellite images) and 2018 – 2022 (drone images).

### 3D model

The drone data were processed using the Agisoft Metashape Professional 1.8.4 to produce a 3D spatial model and orthomosaics. Orthomosaic images of Nov/2018 and Nov/2022 were utilized for the spatial-temporal analysis. A high resolution dense point cloud was executed according to point clouds with point spacing between 4 and 5 cm. The dense point clouds were analyzed to identify points representing the terrain elevation to obtain a Digital Terrain Model (DTM). The DTM corresponds to the ground devoid of vegetation. Another procedure produced a digital surface model (DSM) indicating the vegetation cover, ground, and water (see Agisoft User Manual for details). The DTMs for the wetland substrate and sandy coastal plain were based on drone survey from Nov/2018 and Nov/2022.

The contrasts of colors and elevations of point clouds permitted the identification of points signifying the vegetation, muddy flats, and the sandy coastal flats. The elevation grid for the DTM was based on the mean dense point cloud to minimize the effects of ripples of the beach barrier on the drone surveys. Elevations were referenced to the Geoid Model EGM2008–5. The vertical differences between GCP and the DTM allowed a quantitative analysis of these models, following the Eq. (2) [10]:

(2) $$Z_{dif}=Z_{DEM}--Z_{grd}$$

where *Z_dif_* = the vertical differences, *Z_DEM_* = the Z value of the 3D dense point cloud, and *Z_grd_* = the *Z* value of the GCPs.

The high tidal level was used to reference the shoreline position, while the dune crest was defined as the maximum surface elevation of the beach barrier. The mean sea level (0 m) was used as a baseline for sediment volume calculations. The above procedures were developed in the Global Mapper version 22.1.2 software to calculate cut-and-fill volumes within a selected area. The volume was calculated by breaking the area and assessed into many small squares, and by using the formula: Volume = Height * Pixel Size. Global Mapper also generates cross-shore profiles in a specified path based on loaded planialtimetric datasets (see 2020 Global Mapper User Manual). A spatial and temporal sequence of these planialtimetric profiles allows us to identify changes in the beach barrier morphology.

## Method validation

Topographic and environmental data were obtained during the fieldwork to validate the planialtimetric data and classifications based on spatial analysis and 3D models. According to the GCPs in Table 1, the *X_dif_, Y_dif_, Z_dif_* values were <16 cm, suggesting a planialtimetric error of ± 16 cm (longitude), ± 6 cm (latitude), and ± 5 cm (elevation) for the 3D models, respectively, based on the aerophotogrammetry by drone. The lowest and highest divergences between the Z_3D_ and *Z_grd_* were obtained in the middle and along the periphery of the drone surveyed area, respectively. This trend reflects the lower overlapping points on the edge of the drone mapped area. Afterwards, the dense point cloud showed more consistent points in the middle than on the border of the 3D model. The central sector of the 3D model, ∼100 m away from the edge, presented a ± 2 cm vertical margin of error. Considering the vertical accuracies of the *Z_grd_* values of the GCPs obtained by a Catalyst GNSS receiver (± 1 cm) and a SLHRL (± 2 cm) are lower than the *Z_dif_* values, a ± 5 cm vertical margin of error was admitted for the 3D models. These results show that DEMs based on aerophotogrammetry obtained from a Phantom 4 RTK drone have a margin of error low enough to dispense GCPs.

**Table 1:** Ground Control Points of the study area with longitude, latitude, elevation, and differences (m) between latitudes, longitudes, and elevation obtained by photogrammetry and those obtained in the field by a topographic survey.

GCP	X/LONGITUDE	Y/LATITUDE	Z/ELEVATION (m)	X_DIF_	Y_DIF_	Z_DIF_
1	−94.737656	29.378398	0.02	−0.002	0.016	0.011
2	−94.745543	29.369953	0.03	−0.028	−0.021	0.036
3	−94.739371	29.365928	0.15	0.133	0.036	0.070
4	−94.731807	29.368008	0.05	0.088	0.044	0.009
5	−94.733060	29.371935	0.03	0.000	−0.002	−0.019
6	−94.739360	29.371197	0.06	0.058	0.052	−0.022
7	−94.740147	29.373952	0.03	0.019	−0.087	0.003
8	−94.741154	29.367971	0.05	−0.392	0.067	−0.014
9	−94.743318	29.367455	0.01	0.142	0.011	0.008
10	−94.743778	29.366329	0.02	0.008	0.039	0.047
11	−94.733976	29.366785	1.15	0.022	−0.020	0.059
12	−94.735381	29.366777	0.299	−0.063	0.018	−0.106
13	−94.734718	29.367857	0.32	−0.157	−0.181	−0.050
14	−94.735961	29.366248	0.32	−0.204	0.018	0.094
15	−94.733570	29.367085	1.13	0.010	0.013	−0.051
16	−94.736578	29.365920	1.40	0.363	−0.003	−0.071

## Conclusion

The DJI Phantom 4 RTK provides a more time-efficient method than the conventional topographic survey in establishing the GCPs, and facilitates the assessment of inaccessible coastal areas due to hurricane impacts. Comparison of DEMs before and after hurricanes allows quantifying shoreline retreat, difference in sedimentary volumes, and identifying areas with erosion and sediment accumulation. Orthomosaics permit the individualization and quantification of vegetation/geomorphological units and damage to urban zones and coastal infrastructures. These data are essential in assessing coastal vulnerability in the face of possible flooding and strategies to mitigate the impacts of climatic extremes on the coast, for instance, by coastal restoration projects.

During the past 5 years, we have published a total of 12 studies using this method to monitor the mangroves and coastal sedimentary dynamics from South and North America in the light of climate change [4], [5], [8], [10], [11], [12], [13], [14], [15], and this method has attracted interests from numerous scholars from across the globe. Thus, we believe this procedure can provide an effective way for post-disaster assessment and coastal landscape survey in a timely fashion.

## Ethics statements

This study does not involve any human subjects or animal experiments.

## Related research article

Yao, Q., Liu, K.B., Fan, D.D., Cohen, M.C.L., Oliveira, P.E., and Rodrigues, E. Eco-morphological Evolution of the Bolivar Peninsula (Texas, U.S.A.) during the Last 2000 Years: A Multi-proxy Record of Coastal Environmental Changes. Quaternary Science Reviews (in review), Elsevier production reference: JQSR-D-22-00613

## CRediT authorship contribution statement

**Marcelo Cancela Lisboa Cohen:** Methodology, Software. **Adriana Vivan de Souza:** Data curation, Visualization. **Kam-biu Liu:** Supervision. **Qiang Yao:** Writing – original draft.

## Declaration of Competing Interest

The authors declare that they have no known competing financial interests or personal relationships that could have appeared to influence the work reported in this paper.

## Data Availability

No data was used for the research described in the article.
